# Six Years (2011–2016) of Mandatory Nationwide Bovine Viral Diarrhea Control in Germany—A Success Story

**DOI:** 10.3390/pathogens6040050

**Published:** 2017-10-18

**Authors:** Kerstin Wernike, Jörn Gethmann, Horst Schirrmeier, Ronald Schröder, Franz J. Conraths, Martin Beer

**Affiliations:** Friedrich-Loeffler-Institut (FLI), Südufer 10, 17493 Greifswald-Insel Riems, Germany; joern.gethmann@fli.de (J.G.); schirrmeier@aol.com (H.S.); ronald.schroeder@fli.de (R.S.); franz.conraths@fli.de (F.J.C.); martin.beer@fli.de (M.B.)

**Keywords:** bovine viral diarrhea, epidemiology, prevalence, diagnostics, ear notch sampling, eradication program

## Abstract

Bovine viral diarrhea (BVD) is one of the most important infectious diseases in cattle, causing major economic losses worldwide. Therefore, control programs have been implemented in several countries. In Germany, an obligatory nationwide eradication program has been in force since 2011. Its centerpiece is the detection of animals persistently infected (PI) with BVD virus, primarily based on the testing of ear tissue samples of all newborn calves for viral genome or antigen, and their removal from the cattle population. More than 48,000 PI animals have so far been detected and removed. Between the onset of the program and the end of 2016, the prevalence of these animals among all newborn calves decreased considerably, from 0.5% to less than 0.03%. The number of cattle holdings with PI animals likewise decreased from 3.44% in 2011 to only 0.16% in 2016. Since a large number of naïve, fully susceptible animals are now confronted with BVD virus, which is still present in the German cattle population, the challenge of the coming years will be the identification of remaining PI animals as quickly and efficiently as possible, and the efficient protection of BVD-free farms from reinfection.

## 1. Introduction

Bovine viral diarrhea (BVD) is endemic in cattle populations worldwide, and causes major economic losses and significant impact on animal welfare [1,2,3]. The causative agent of the disease, a pestivirus within the family *Flaviviridae*, exists in the two species BVDV-1 and BVDV-2, and is closely related to the ovine Border disease virus and the classical swine fever virus [4]. According to their growth in cell culture BVDV isolates are classified into the two distinct biotypes cytopathic (cp) and non-cytopathic (ncp). Contact of naïve, non-pregnant cattle with BVDV results, in most cases, in clinically inapparent infections, or leads to mild or moderate unspecific symptoms like diarrhea, fever, reduced milk yield, hemorrhagic lesions, or pneumonia [5]. The immunosuppression induced by the virus can promote secondary infections, which regularly lead to an impairment of the general health conditions on a herd level. Occasionally, severe acute forms of BVD occur, which are characterized by hemorrhagic syndromes and mucosal disease-like lesions, and are mainly associated with virulent BVDV-2 strains [6].

An infection of naïve, pregnant cattle with BVDV may depend on the phase of gestation, result in abortion, stillbirth, or teratogenic effects. When the infection occurs during the first trimester, it may cause the birth of immunotolerant, persistently ncp BVDV-infected viremic calves, also known as persistently infected (PI) animals [7]. These PI animals are immunotolerant to the BVDV they are infected with, and are therefore unable to develop specific antibodies against this particular virus strain. As a consequence, the animals shed enormous amounts of BVDV throughout their lives, which makes them the major source of the spread and perpetuation of BVDV within individual herds, as well as for transmission to cattle holdings previously not affected by BVD [8,9]. The inevitably fatal mucosal disease (MD), which develops only in PI cattle, is associated with the superinfection of the PI animal with a cp-strain that is antigenically homologous to the persisting ncp-strain. Another possibility is the appearance of a cp-biotype arising from mutations of the ncp-BVDV already circulating in the animal [10]. Clinical signs of this late-onset form of BVD include severe and bloody diarrhea, mucosal lesions, and rapid wasting [10,11].

Due to their major importance in intra- and inter-herd virus spread, PI animals are the main target of all efforts to reduce clinical diseases and BVD-induced economic losses. However, despite the common goal of BVDV-elimination from the respective cattle population, different approaches have been selected for eradication programs that have been implemented during the last decades in several European countries [12,13,14]. The Scandinavian model, which has also been implemented in Lower Austria, was based on large-scale bulk milk serology combined with a strict non-vaccination policy to preselect farms with an elevated risk for the presence of PI animals. Subsequently, all animals from these herds were tested individually, detected PI animals removed, and biosecurity measures, in combination with ongoing serological monitoring, established. Under the Scandinavian conditions, freedom from BVD was achieved within a timeline of about 10 years [13,15].

An alternative approach to BVD control, which proved beneficial especially for countries with a high initial BVDV prevalence and a high level of cattle trading and transport combined with ongoing vaccination campaigns, was based on the direct antigen or viral genome testing of all animals without serological pre-screening. In 2008, Switzerland started a three-step program without vaccination following this approach [16], and the prevalence of PI animals decreased from 1.3 to 0.02% within 5 years [17]. The detection and removal of PI animals was further supported by trade restrictions for farms with BVDV-positive animals. Ireland chose the same approach. In 2012, the country implemented an eradication program on a voluntary basis, followed in 2013 by an obligatory program focusing on the testing of ear notch samples of all newborn calves [18,19].

## 2. The German BVD Control Strategy

In Germany, BVD/MD has been a notifiable disease since 2004. A voluntary control program had already started in 1998 in the responsibility of the individual federal states [12]. However, within a timeframe of 10 years, no or only moderate progress was made by this voluntary campaign. As a consequence, a consistent nationwide BVD eradication program was implemented. In December 2008, a regulation for an obligatory control program (Verordnung zum Schutz der Rinder vor einer Infektion mit dem Bovinen Virusdiarrhoe-Virus; http://www.gesetze-im-internet.de/bvdvv/) was decreed by the Federal Ministry of Food and Agriculture, which came into force on the 1st of January 2011. The defined major objective is the fast and efficient reduction of the prevalence of PI animals, and the establishment of farms with a status certified as keeping only “BVD-unsuspicious” (=virus free) animals. In the German legislation, a PI animal was defined as an animal that tested positive for BVDV antigen or genome twice with a maximum interval between both samplings of 60 days (40 days since June 2016), or that tested positive only once without a confirmatory test. The follow-up tests are allowed to differentiate between persistently and transiently infected animals. Furthermore, the offspring of PI animals are also regarded as PI, as well as animals that suffer from MD. Dams that had given birth to a “BVD-unsuspicious” calf were declared likewise “unsuspicious” (=derived BVD status) on the basis of entries on the BVD status of the respective animals in the German cattle database (HI-Tier).

In Germany, approximately 12.4 Mio heads of cattle are kept, about 75% of them in holdings with more than 100 cattle, which represent only 26% of the holdings. On the other hand, about 56% of the holdings keep fewer than 50 cattle (source: Destatis [20]). The average herd size varies between the federal states; while in eastern Germany, 90% of the cattle are kept in holdings with more than 100 animals, the percentage of animals kept in holdings of that size in southern Germany is only about 50%. Due to these size differences in cattle holdings, traditional regional differences in agricultural and trading structures and the generally high, but varying BVD prevalence, the national eradication program adopted the Swiss approach of testing all newborn calves for the presence of BVDV antigen or genome. At the beginning of the nationwide control program, four basic rules were defined: (1) mandatory testing of all newborn calves within the first 6 months of life for BVDV antigen or genome; (2) immediate elimination of all detected PI animals; (3) trade only with “BVD-unsuspicious” animals (including quarantine in affected farms); and (4) prevention of reinfections by qualified measures, such as implementation of biosecurity measures or vaccination. In contrast to most European countries with BVD eradication programs, voluntary vaccination is permitted in Germany; several inactivated vaccines, either as monovalent preparations or in combination with immunogens against further pathogens, as well as two live vaccines, are licensed. To induce a long-lasting immune response, and to ensure fetal protection, a two-step immunization scheme based on inactivated formulations for priming and live attenuated vaccines for booster vaccination is recommended [21,22].

As indicated in the regulation, BVD diagnostics have to be carried out with test systems prescribed in the German Official Collection of Test Methods for BVD [23]. In Germany, about 4.8 million calves are born per year (source: HI-Tier); combined with follow-up tests and the investigation of older or imported animals, nearly 5 million diagnostic BVDV tests are performed every year. The vast majority of tests are carried out using ear biopsies as the sample material, which are taken during the tagging procedure that has to be performed for every calf in the European Union within the first seven days of life. In addition to ear notch samples, blood samples are investigated, primarily for confirmatory testing. However, the use of this sample material from newborn calves is very limited, due to the strong inhibitory effect of maternal antibodies (“diagnostic gap”) [24,25,26,27]. For routine diagnostics in the local veterinary state and private laboratories, both, E^rns^-based antigen ELISA and real-time RT-PCR, are applied. Commercial tests for BVD diagnostics require the official approval of the Friedrich-Loeffler-Institut, Germany’s Federal Research Institute for Animal Health, since licensing and batch release are part of the quality assurance scheme for diagnostic systems used for notifiable and reportable animal diseases (according to § 11 Abs. 2 TierGesG). The licensed and released tests have to be carried out according to the approved instructions.

All test results are entered in the German cattle trade database (HI-Tier), and within the database, an algorithm calculates the status of the animals. For this study, the classification, the number of newborns per year and federal state, and the number of farms keeping cattle in the respective period were extracted from HI-Tier. The number of PI animals was set in relation to the number of newborn animals, and the percentage of affected farms was calculated from the number of farms that had at least one PI animal during a certain period in relation to all farms keeping cattle in this period.

## 3. Progress of BVD Eradication 

Between the start of mandatory testing in 2011 and 2016, more than 48,000 PI animals were removed from the German cattle population. The proportion of animals classified as PI among all newborn calves was considerably reduced from 0.5% (23,792 PI among 4,929,160 newborn calves) to less than 0.03% (1005 PI among 4,915,421 calves), whereby nearly a halving of the PI prevalence was observed each year [28]. However, variations in the implementation of control and flanking measures exist, because animal disease control lies in the responsibility of the German federal states. Furthermore, the starting prerequisites in 2011 differed between the federal states, since some states implemented voluntary programs and/or started with ear notch testing prior to 2011.

**Figure 1:**
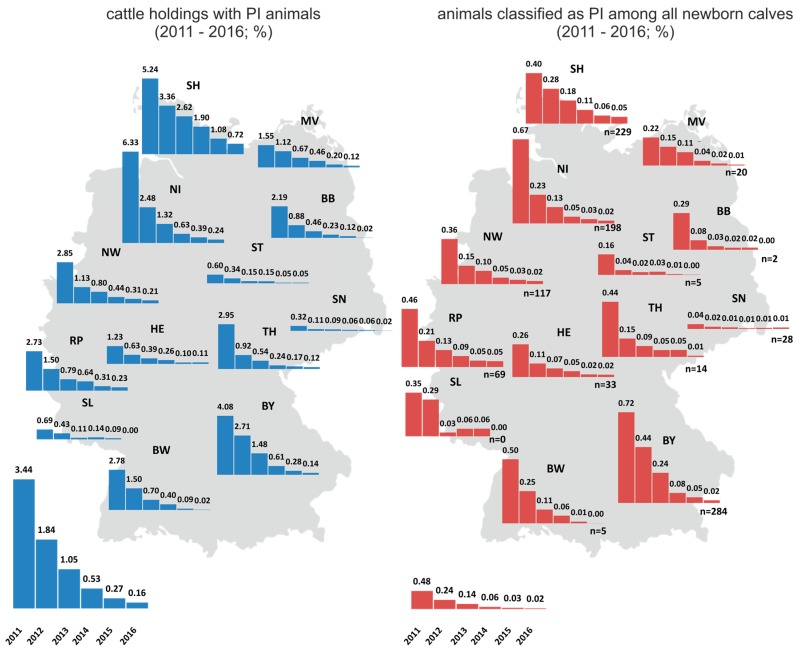
Cattle holdings with PI animals (**left**) and PI animals among all newborn calves (**right**) per German federal state. The prevalences for 2011 to 2016 are shown individually for each federal state, and the chart is placed at the corresponding position on the map of Germany. The overall prevalences for Germany are shown under each map. n—numbers of PI animals.

Variations in the implementation in individual federal states, in combination with the aforementioned differences in cattle farming structures, also led to differences in PI prevalence between federal states (Figure 1). While, for instance, in Schleswig-Holstein, the proportion of PI animals among all newborn calves was approximately 0.05% (n = 229) in 2016, in e.g., Brandenburg, Saxony-Anhalt, or Baden-Württemberg, fewer than 10 PI animals were born in the same year (Figure 1). The number of cattle holdings with PI animals likewise decreased considerably (Figure 1). While PI animals had been found in more than 8000 farms in 2011, only 324 holdings were affected in 2016. This implies that in more than 99.8% of all German cattle holdings no PI animal was detected in that year, proving the very high efficiency of the control program.

However, the generation of an increasingly naïve cattle population creates new challenges, such as an elevated risk for re-infections, as observed during the 2012/13 BVDV-2c epidemics in western Germany [29,30]. This emphasizes the necessity and the impact of stringent biosecurity measures, which must become an integral part of the BVD control program. Due to the intended efficient and continuous elimination of PI animals, the number of BVDV antibody-negative animals has increased in recent years. Although seroprevalence studies for Germany as a whole are missing, regional studies conducted in collaboration with the Friedrich-Loeffler-Institut suggest that the seroprevalence may range between 10% and 25%, with regional differences, which depend among other things, on the regionally differing usage of vaccines. Therefore, a large number of naïve, susceptible animals are confronted with the field virus, which is still present in the German cattle population. In addition, the prevalence curve seems to decrease asymptotically towards zero, which was expected, but may still lead to a stagnation in the eradication campaign or drawbacks in the future, if the stringency of the program, the awareness or the motivation of farmers and stakeholders regarding the need for the BVD control decline. Thus, there is an urgent need for a continued analysis of risk factors, which may endanger success, and must therefore be addressed by adjustments of the control measures.

## 4. Gaps in the Regulation and Amendment

With the experience of the first years of the German nationwide mandatory BVD eradication program, the following risk factors were identified, which could negatively influence the progress of the program:

- Long possible evaluation period of the newborn calves until the age of 6 months
- Keeping PI animals, often for weeks or months, on the farms, although immediate elimination is compulsory unless permission for re-testing is granted
- Missing or insufficient biosecurity measures, and deficiencies in management systems and gaps in risk awareness
- Contact to animals with unknown status during exhibitions, livestock markets, fair and animal trading points or on common pastures
- Trade with untested calves, e.g., for export
- Import of cattle with uncertain BVD status
- Derogations for beef cattle
- Lack of restrictions for cattle holdings with PI animals
- Underestimation of transiently infected cattle, especially so-called “Trojan” cows (= pregnant dams carrying a PI-fetus, but not PI themselves)
- Insufficient success control by serological monitoring
- Imperfect test systems (sensitivity not 100%)
- Lack of supranational regulations and guarantees in the European Union as for example implemented for bovine tuberculosis, brucellosis, enzootic leucosis and bovine herpesvirus type 1 (directive 64/432/EG)

Based on these identified gaps and risks, the German BVD regulation was adjusted in June 2016. The major goal of the modifications was the identification of the remaining PI animals as quickly and efficiently as possible, and to remove them from the cattle population. For this purpose, some deadlines were shortened, and restrictions for animal holdings with PI animals were implemented. The central adjustments and modifications of the BVD regulation include:

- Shortening of the evaluation period of newborn calves from 6 months to the first month of life
- Shortening of the maximum interval between initial and confirmatory samplings from 60 to 40 days
- Immediate elimination of all detected PI animals (slaughter is permitted)
- Movement restrictions for farms with PI animals: 40 days for every unvaccinated cattle and for pregnant dams until birth to avoid the movement of “Trojan” cows to unaffected holdings
- Possibility of serological stock monitoring by milk serology or spot-testing of young stock (calves > 6 months of age to avoid the influence of maternal antibodies) in addition to ear notch testing

The aforementioned adjustments take the identified or presumed gaps and the requirements to secure the achievements made so far into account and should ensure the further progress towards a cattle population free of BVDV in the nearer future. The elimination of PI animals as the main element and the primary testing of ear tissue samples are retained. In addition, first serological tools are permitted, and will be evaluated in pilot studies regarding their suitability for BVD monitoring in the field, thus forging the bridge from BVD eradication to surveillance of a BVD-free status.

With the implementation of the adjustments, the prevalence of PI animals should further decrease, thus enabling in the foreseeable future the replacement of ear notch testing by serological methods such as spot-testing or bulk milk serology, as was demonstrated by countries with well-advanced control programs [17]. However, the possibility of implementing such serological screening methods is related to the extent of vaccination. When bulk milk serology is used to monitor possible new introductions of BVDV into unaffected herds, a vaccination ban has to be discussed, at least until marker vaccines are available that allow differentiation between vaccinated and field-infected animals. Furthermore, the success of the program is highly dependent on the strict and systematic implementation of biosecurity measures, which represent a central element of BVDV control [13], combined with a high level of awareness and motivation among farmers and stakeholders.

## 5. Lessons to Be Learned

During recent decades, BVD control approaches in various European countries and regions have clearly demonstrated that measures on a voluntary basis are inadequate to achieving freedom from the disease. Countries that started with such an approach eventually switched to a consistent, mandatory program. By this means, several countries, including Norway, Sweden, Finland, and Denmark, have successfully eradicated the disease, or are now almost free from BVD. Further countries, e.g., Switzerland, Austria or Germany, are in an advanced or final stage of BVD eradication, while some, including the Netherlands and Ireland, only recently implemented control programs [3,18,31,32,33,34]. The experiences gained in Europe suggest that both basic approaches, i.e., the Scandinavian model and PI detection and removal based on ear notch testing, are suitable for achieving considerable success [14]. These control options promise considerable savings compared to the costs caused by BVD infections [35].

In summary, the obligatory German BVDV control program is a success story like other comparable programs carried out in Europe thus far. However, drawbacks are possible in all phases of the program. They can often be attributed to international trade with cattle of uncertain BVD status, missing or insufficient biosecurity, and management practices on farms and in cattle trade. The situation is further complicated by the lack of a concerted EU-wide BVD control strategy [3] and accompanying regulations, although these exist and have been successfully applied to prevent the spread of other animal diseases, such as bovine tuberculosis, brucellosis, enzootic leucosis, and bovine herpesvirus type 1 at the supranational level (directive 64/432/EG). Therefore, transnational rules and measures would be beneficial, if not necessary, but they are difficult to establish.
